# Redox-Mechanisms of Molecular Hydrogen Promote Healthful Longevity

**DOI:** 10.3390/antiox12050988

**Published:** 2023-04-24

**Authors:** Md. Habibur Rahman, Eun-Sook Jeong, Hae Sun You, Cheol-Su Kim, Kyu-Jae Lee

**Affiliations:** 1 Department of Convergence Medicine, Wonju College of Medicine, Yonsei University, Wonju 26426, Republic of Koreacs-kim@yonsei.ac.kr (C.-S.K.); 2 Department of Anesthesiology & Pain Medicine, Anam Hospital, Korea University College of Medicine, Seoul 02841, Republic of Korea

**Keywords:** aging, molecular hydrogen, reactive oxygen species, oxidative stress, therapeutic effects, redox mechanism, antioxidant, longevity

## Abstract

Age-related diseases represent the largest threat to public health. Aging is a degenerative, systemic, multifactorial and progressive process, coupled with progressive loss of function and eventually leading to high mortality rates. Excessive levels of both pro- and anti-oxidant species qualify as oxidative stress (OS) and result in damage to molecules and cells. OS plays a crucial role in the development of age-related diseases. In fact, damage due to oxidation depends strongly on the inherited or acquired defects of the redox-mediated enzymes. Molecular hydrogen (H_2_) has recently been reported to function as an anti-oxidant and anti-inflammatory agent for the treatment of several oxidative stress and aging-related diseases, including Alzheimer’s, Parkinson’s, cancer and osteoporosis. Additionally, H_2_ promotes healthy aging, increases the number of good germs in the intestine that produce more intestinal hydrogen and reduces oxidative stress through its anti-oxidant and anti-inflammatory activities. This review focuses on the therapeutic role of H_2_ in the treatment of neurological diseases. This review manuscript would be useful in knowing the role of H_2_ in the redox mechanisms for promoting healthful longevity.

## 1. Introduction

Almost all major human diseases, including atherosclerosis, cancer, cardiovascular disease, metabolic syndrome, dementia, hypertension, and other neurodegenerative diseases have aging, a biosocial concern, as their underlying basis. To help older people maintain their health for as long as possible and to deal with an ever-increasing population, it is essential for healthcare providers to improve the prevention and control of age-related disorders. Diet is a useful and reasonably priced approach to helping seniors live longer and healthier lives [1]. Aging is seen mainly in protected settings as an evolving phenomenon that enables longevity in the wild beyond the normal lifespan. Aging is characterized by the accumulation of nucleic acids, proteins and lipids formed as a result of molecular damage. The free-radical rationale of aging has long been established among the theories explaining the aging process [1]. Aging occurs when several defense mechanisms fail to respond to the damage caused by reactive oxygen species (ROS), particularly in the mitochondria [2]. The key causes of aging-induced damages are the ineffectiveness and inability of the maintenance, repair, and turnover pathways [3]. Aging is related to the propensity for adverse water balance and makes older subjects more vulnerable to dehydration [4]. Water accounts for approximately half the weight of the human body and is necessary for human life and health [5,6]. There is growing evidence that even mild dehydration (determined to be 1–2% body mass loss due to fluid deficit) may lead to various age-related diseases, including arthritis, cataracts, osteoporosis, type 2 diabetes (T2D), hypertension and Alzheimer’s disease (AD) [7]. The type of water supplied as drinking water plays an important role in determining the safety and health issues because tap water quality continues to cause public concern [8,9], with some countries demanding the derogation from European water quality standards [10]. Groundwater is the Earth’s most abundant and important freshwater resource [11]. Although dietary food components have been shown to improve cognitive function in older people [2], the effects of different nutritional compounds on other biomarkers of aging are much less understood. A dietary supplement, which has a direct impact on telomere metabolism, slows telomere decline and reduces aging and might expand life and improve health [3]. Nowadays, molecular hydrogen (H_2_) can be used safely in the air at body temperature and at concentrations less than 4.7%. H_2_ selectively quenches toxic ROS and has an anti-apoptotic, anti-oxidant, anti-inflammatory and anti-allergic impact, and it has become a new non-oxidant [12]. H_2_ has recently been studied in preclinical and clinical studies under various conditions linked to oxidative and inflammatory stress, such as heart failure due to radiation, ischemia-reperfusion (I/R), myocardial infarction, brain infarction, heart storage and heart transplants [13]. Hydrogen-rich water (HRW) has recently come to light as a novel dietary beverage that may improve several aging-related characteristics in interventional trials, reducing different inflammatory responses that may help prevent programmed cell death [4], improve nutrient metabolism, repress wrinkle formation and increase other physiological activities [5]. Japanese centenarians were discovered to have higher levels of H_2_ gas, which indicated that the intestinal production of H_2_ gas may have conferred upon them longevity and reduced oxidative stress [6,7]. Increase in longevity was also reported by another study, stating intestinal production of H_2_ gas as the apparent reason [8]. Cardiovascular and oncological disorders, the primary cause of morbidity and mortality worldwide, are more than 93% [14,15]. Pathological disorders, such as cardiac fibrosis, liver injury, neuronal diseases, and diabetes, causally involving free radicals have been investigated for the protective effects of H_2_ [13]. Ischemia and subsequent heart reperfusion are other disorders in which a large number of tissue-damaging free radicals are formed [16]. One study revealed that drinking HRW for 6 months favorably affected different age-related features, including general pain, telomere strength and brain metabolism—indices that helped to increase anti-oxidant activity; HRW also enhanced sleep quality [9]. It has been reported that increased H_2_ gas generation in the intestine depends on the presence of undigested carbohydrates and hydrogen-producing bacteria that are affected by some environmental conditions [10,11]. Over a thousand peer-reviewed study papers have been published thus far, demonstrating the wide-ranging interest in H_2_ biomedical research.

In this review, we highlight the emerging role of H_2_ in the prevention of age-related diseases, Alzheimer’s, Parkinson’s, cancer and osteoporosis, etc. This review manuscript would be useful in knowing the role of H_2_ in the redox mechanisms for promoting healthful longevity.

## 2. The Mechanism of Action of H_2_

H_2_ suppresses the allergic [12] and inflammatory signaling pathways [13]. The anti-oxidative stress effect of H_2_ was initially thought to be conferred upon by the direct elimination of hydroxyl radicals and peroxynitrite. Subsequent studies reported that H_2_ activated the system nuclear factor erythroid 2–related factor 2 (Nrf2) [14,15] and its downstream heme oxygenase-1 (HO-1) [16]. Kawamura et al. (2013) suggested that H_2_ in Nrf2-knockout mice did not relieve hyperoxic lung injury [17]. In addition, at the Medical H_2_ Symposia in 2012 and 2013, Ohsawa et al. (2012) stated that H_2_ enhanced mitochondrial functions and induced Nrf2 nuclear translocation. Furthermore, Matsumoto et al. reported that oral H_2_ water intake increased ghrelin gastric expression and secretion and that the ghrelin receptor-antagonist and ghrelin secretion-antagonist abolished the neuroprotective effects of H_2_ water [18]. At the 5th Medical H_2_ Symposium in Nagoya, Japan, in 2015, Ohta et al. demonstrated that H_2_ affects the free radical chain reaction of unsaturated fatty acids on the cell membrane and modifies the lipid peroxidation process [12]. This irregular oxidation of the phospholipids at low levels of H_2_ (at least 1.3%) has also been reported indicating that the biological effects of H_2_ can be explained by the aberrant oxidation of the phospholipids upon exposure to H_2_. Among the many molecules altered by H_2_, most molecules were predicted to be “passengers” (downstream regulators), secondarily modulated by the pilot (master regulator) [12]. Proving the effect of H_2_ in an in vitro setting would be the best way to classify the master regulator. While nothing is known about the lipid peroxidation analysis, the second master control body for H_2,_ next to the radical scavenging effect, may be the free chain response to lipid peroxidation [12]. Intracellular signal transduction systems are modulated by H_2,_ and the downstream gene expression is regulated to alleviate disease processes. Moreover, biologically active substances, which modulate signaling molecules, damage our bodies [19]. Inhaling a gas mixture containing H_2_ (less than 4%) is effective in protecting against acute oxidative stress, according to one research study [20]. Another research study showed that it is safe and more practical to dissolve H_2_ in water up to 0.8 mM under atmospheric pressure at room temperature [21]. H_2_ also mitigates surgery-induced cognitive impairment [22]. Following 4% H_2_ inhalation, the liver’s H_2_ concentration rose quickly and reached balance in about 5 min at 20 mol/L. HRW consumption resulted in sporadic availability to H_2_. One study revealed that, even after 8 h, supersaturated H_2_ in HRW (1000 mol/L) was maintained at a high content and was still above 600 mol/L [23].

Remarkably, the effects of saturated HW were virtually identical to those of H_2_ concentrations as low as 0.08 ppm. (1.5 ppmH_2_). Within 30 min of consuming HW, the majority of H_2_ in the blood is invisible [24]. Another example would be that the amount of H_2_ exposed to a 60-kg individual for 24 h as a 2% gas would be at least 104 times greater than what would be consumed by drinking saturated HW. However, HW is sometimes even more efficient than H_2_ at achieving its goals [25]. Drinking H_2_-rich water reduced fatigue in healthy people, according to one research [26]. Additionally, blood flow-dependent vasodilatory responses in people were enhanced by H_2_-rich water [27]. In radiotherapy patients with liver cancer, it helped appetite and taste issues and reduced oxidative stress in the blood [28]. It was reported that H_2_-rich water improved cognitive impairment [29]. In addition, drinking H_2_-rich water improved neuropsychiatric and endocrine metabolic disorders in vivo study [29].

## 3. The Anti-Oxidative Effects of H_2_ That Extend Life Span

Although H_2_ has long been assumed to be an inert gas for living organisms, an animal study found that owing to its anti-oxidant properties, inhalation of H_2_ gas reduced oxidative stress and stifled the brain damage caused by I/R injury. Among several proposed biological activities, the function of H_2_ as an anti-oxidant has received the greatest attention. Furthermore, even after elimination of H_2_ from the body, especially at low concentrations, its biological and anti-oxidant benefits continue to exist, implying that the mechanism may include modulation of anti-oxidant signaling rather than actual free radical scavenging [24]. H_2_ is a specific scavenger of hydroxyl radical and peroxynitrite, powerful oxidants that interact without distinction with nucleic acids, lipids and proteins leading to DNA breakage, lipid peroxidation and protein inactivation [25,26]. In both human diseases and rodent models, H_2_ administration reduces the expression of various oxidative stress markers, such as myeloperoxidase, malondialdehyde (MDA) and 8-hydroxy-desoxyguanosine (8-OHdG) [27,28]. In addition, H_2_ can also minimize myeloperoxidase expression [29], decrease the function of mitochondrial oxidoreductase and stabilize the mitochondrial membrane potential to reduce the tissue damage caused by oxidative stress [30]. In 2016, researchers proposed that H_2_ may reduce the ROS content depending on the endogenous glutathione peroxidase in *Ganoderma lucidum* [31]. Another study demonstrated that HRW intake affected different aging-related characteristics in aged people, such as extension of telomeres and improvement in DNA methylation [9]. Several studies have shown that H_2_ is not cytotoxic even at high concentrations [32,33]. H_2_-water consumption reduced the development of oxidative stress and avoided cognitive decline; therefore, it can play a role in extending the life span [34]. In rats, H_2_-water stopped the onset and spread of nigrostriatal degeneration [35]. Numerous studies have shown that H_2_ reduces apoptosis during the treatment of septic injury in rodents [36,37]. Many studies have demonstrated that H_2_ reduces ROS, increases anti-oxidant enzyme activity and inhibits pro-oxidant enzyme activity to mitigate the tissue damage caused by lipopolysaccharides [38].

## 4. The Anti-Inflammatory Effects of H_2_

A study reported that H_2_ breathing capacity could cure liver inflammation caused by parasites and was the first to demonstrate the anti-inflammatory properties of H_2_ [39]. Hydrogen has been shown to exhibit anti-inflammatory activity in multiple injury models. H_2_ is known to prevent the oxidative stress-induced inflammatory tissue damage by downregulating pro-inflammatory and inflammatory cytokines [40], such as interleukin (IL)-1β, IL-6 and tumor necrosis factor-α (TNF-α) [41]. H_2_ can also drastically decrease NF-kB expression post-liver damage [42]. In animal models of allergic rhinitis or I/R cerebral injury, H_2_ also has anti-inflammatory effects via upregulation of regulatory T cells (Tregs), which have an immunosuppressive effect and reduction in the expression of NF-kB [43]. Similarly, a study found that increasing the expression of the heat stress protein Hsp60, which is stimulated at high temperatures to protect itself, may successfully prevent acute pancreatitis in mice in the early stages through pre-inhalation of H_2_ [44].

## 5. H_2_ and Redox Mechanism of Oxidative Stress

**Figure 1:**
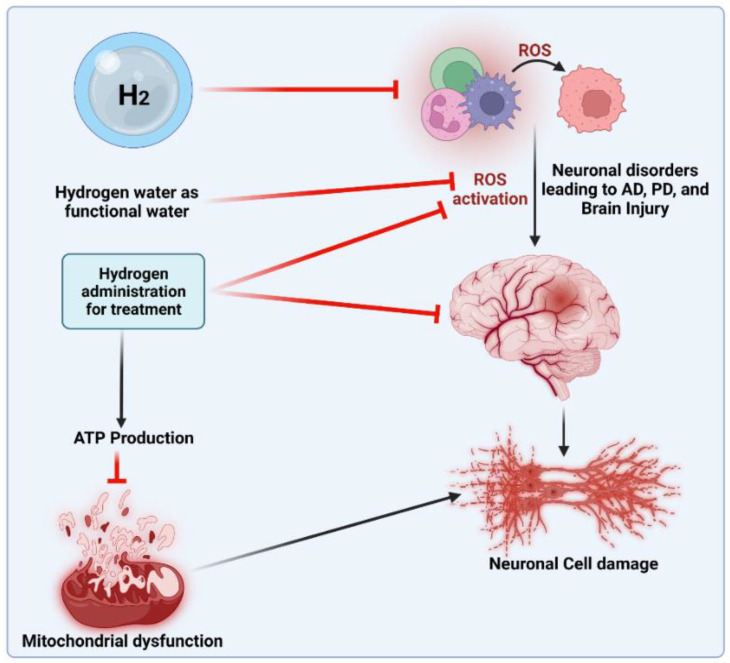
Protective effects of H_2_ in treating different age-related diseases.

The anti-oxidant effects of H_2_ are primarily expressed in certain ways. First, H_2_ was discovered to specifically eliminate hydroxyl radicals and peroxynitrite. H_2_ can readily penetrate biofilms compared to standard anti-oxidants, such as superoxide dismutase (SOD), catalase and alpha-tocopherol, and does not influence the usual metabolic redox reaction, owing to its small molecular weight and anti-oxidant activity, which selectively affects only the strongest oxidant [45]. H_2_ can also directly downregulate ROS or act as a gas-mediated signal regulator. Recently, a study [46] showed that H_2_ in the urine, a marker of oxidative stress, can increase quickly and approximately to the same level as that induced by exercise. During cell adaptation, the production of exercise-induced ROS is necessary, and short-term ROS exposure can protect neurons from oxidative stress [47]. H_2_ can mediate beneficial effects of the mitohormetic effectors of hormone processes on the body [46]. However, the anti-oxidative mechanism of H_2_ may affect the free radical chain reaction of lipid peroxidation. Many studies have shown that H_2_ protects cells by preventing the peroxidation of lipids and fatty acids [48]. According to wear-and-tear theory, aging is the slow deterioration of the body’s cells and tissues due to oxidative stress, radiation exposure, exposure to toxins or other deteriorating processes [49]. Denham Harman [50] introduced the free radical theory of aging [51] in the 1950s. Numerous studies have shown that oxidative damage and ROS levels rise with aging [52], that reducing oxidative damage increases lifespan in a variety of model organisms (such as yeast, nematodes, fruit flies, mice, etc.) and that both higher ROS production and oxidative damage have detrimental effects on lifespan [53]. In addition, H_2_ can reduce myeloperoxidase expression [46], decrease mitochondrial oxidoreductase activity [54] and stabilize mitochondrial membrane potential [55], thus enhancing tissue damage resulting from oxidative stress. The protective mechanism of H_2_ in treating different age-related diseases is shown in Figure 1.

## 6. Age-Related Diseases and Redox Mechanisms

**Figure 2:**
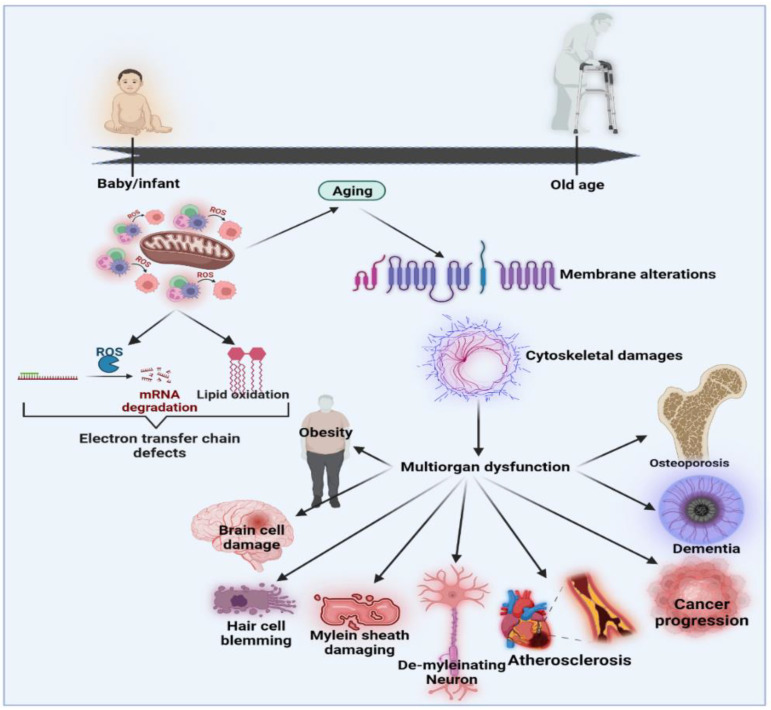
Effects of oxidative stress and the associations between aging and age-related diseases modified from [56].

Over the last few decades, several models have been suggested to define the relationships and biopathways of aging [56]. The generally accepted theory is the “oxidative stress hypothesis”, which advances and improves upon the free radical aging theory [57,58]. The oxidative stress theory underlines the crucial role of anti-oxidant defenders in the overall redox balance [59]. Ito et al. (2011) performed an open-label H_2_-water analysis (1.0 L/day) for 12 weeks in 14 patients with muscle disorders, including muscular dystrophy and mitochondrial myopathies. This open-label research showed significant improvements in the lactate: pyruvate ratio, fasting blood glucose, serum matrix metalloproteinase-3 (MMP3) and triglyceride levels [60,61]. In mitochondrial myopathies, the lactate: pyruvate ratio, a responsive biomarker of a weakened mitochondrial electron transportation system, decreased by 28%. In addition, MMP3, the marker of inflammation, decreased by 27% in dermatomyositis. Then, for eight weeks, 22 people with dermatomyositis and mitochondrial myopathies were recruited for a randomized double-blind, placebo-controlled, crossover H_2_-water or placebo dehydrogenated water (0.5 L/day) examination [60]. H_2_ may provide an interpretation of multiple energy booster advantages seen in H_2_ intervention studies that are not due to H_2_ but which do not control the growth hormone secretagogue receptor (GHS-R1α) in tissues rich in mitochondria (including breast, skeletal muscle, myocardium, testis or liver) [62]. Mitochondria are the most important organelles responsible for energy production via oxidative phosphorylation, which is essential for cellular behavior and adenosine triphosphate (ATP) generation [63]. The formation and oxidation of ROS occurs under normal, healthy conditions in a regulated manner. Changes in the redox state and immune system dysregulation may result in increased systems inflammatory status during aging. Both processes induce inflammatory mediators to stimulate redox imbalances through oxidative stress [64]. The net effects of poor protection by anti-oxidant systems and aggression by reactive species, such as superoxide, hydroxyl radicals, peroxynitrite and H_2_ peroxide, are most likely to cause age-related redox imbalances [65,66]. Functional shifts may be seen as pathophysiological connections to degenerative disorders correlated with age and unresolved chronic inflammation throughout aging [67]. The functional activities of certain proteins require certain prosthetic groups to be covalently connected to the polypeptide chain. These normally involve the conversion of inactive apoproteins into enzymes through complex organic molecules that, for example, engage in protein activity. In addition, some of the posttranslational changes influence biochemical processes through different enzyme operations [68]. To improve homeostatic cell operation, the preservation of a healthy redox balance is essential for a physiological acid-base buffer system in the body. Modernization of the redox balance would greatly impact transcription and mobile signal pathways because most activations and reactions rely on reduction/oxidation processes [55]. Figure 2 shows the effects of oxidative stress and the associations between aging and age-related diseases [56].

## 7. Effects of Hydrogen Gas on Inflammatory Cytokines

Inflammatory cytokines affect a number of signals, which mediate an innate immune response and can aid dysregulation in many diseases, including cancer [69,70]. Common inflammatory cytokines include white blood cell-secreted ILs and macrophage-secreted TNFs, both of which have been closely correlated with cancer initiation and progression [71,72]; both ILs and TNFs can be blocked by H_2_ gas [73]. In cancer patients, chemotherapy-induced inflammation not only causes adverse events [74,75], it also promotes cancer metastasis and treatment failure [76]. By regulating inflammation, H_2_ gas may prevent tumor development, progression and decrease the side effects of chemotherapy and radiation therapy [73].

## 8. Hydrogen Gas Relieves Adverse Effects of Chemotherapy

The leading methods for treating cancer are chemotherapy and radiotherapy [77,78]. However, cancer patients are frequently fatigued, and their quality of life is compromised [79,80]. During cancer, the generation of ROS skyrockets and contributes to adverse outcomes, which lead to severe oxidative stress and inflammation [81]. Therefore, H_2_ gas, on account of its anti-oxidant, anti-inflammatory and other cell-defensive characteristics, can be used to suppress these adverse effects. Doxorubicin, a fatal dilated cardiomyopathy and hepatotoxicity causing antibiotic, is also an important cancer antibiotic used in the treatment of various cancers [82,83]. An in vivo study showed that intraperitoneal injections of saline rich in H_2_ decreased mortality and doxorubicin-led cardiovascular dysfunction. H_2_ rich water has also been shown to exert renal protective effect against cisplatin-induced nephrotoxicity in rats. Treatment with hydrogen rich water can significantly reverse the toxic effects, and it demonstrated a significantly higher rate of cross-relation by the removal of oxygen radicals [84,85]. In another study, the inhalation of H_2_ gas (1% hydrogen in air) and the use of water rich in hydrogen (0.8 mM Hydrogen in water) reversed the body-weight loss and the mortality caused by cisplatin due to the antioxidant property of H_2_ [73]. Similar findings were also reported by Meng et al. (2015), who showed that hydrogen-rich saline could mitigate follicle-stimulated hormone release, increase estrogen levels, improve follicle growth and reduce cisplatin-induced ovarian cortex damage [86]. In a previous study, cisplatin induced higher oxidation levels during therapy and suppressed the activity of antioxidant enzymes. In another study, a six-week intake of H_2_ rich water in patients with malignant liver tumors minimized reactive oxygen metabolites and increased antioxidant activity [87]. Remarkably, the quality of life during radiotherapy was found to be greatly improved in the H_2_-rich water consuming group in comparison to the placebo groups. Both groups showed similar tumor reactions to radiation therapy, indicating that the ingestion of water rich in H_2_ decreased the oxidative stress due to radiation without undermining the antitumor effect of radiation therapy [87]. The various routes of administration, application and mechanisms of action of H_2_ molecules in cancer treatment are listed in Table 1.

## 9. Hydrogen and Intestinal Microbiome

In recent years, gut microbiota brain axis (GMBA) has been recommended as an important therapeutic target for neurological disorders affecting the central nervous system, such as AD [97,98]. Several mechanisms play a key role in preventing bacterial overgrowth in the proximal gut, including migrating motor complex, gastric acid, gut immune system and biliary secretions [99]. During fermentation, H_2_ is produced in the large intestine; this may be excreted through the breath and flatus or metabolized by the flora [100]. Moreover, the proportion of H_2_ excreted in the breath varies depending on its production rate. In addition, the fermentation of lactulose generated more H_2_ than that generated by resistant starch or pectin. HRW is a promising functional drink with positive effects on human health. Over the past decade, the publication of approximately 150 papers related to HRW in human trials, have shown multiple advantageous effects of HRW consumption [101]. According to a study, H_2_ delivered by HRW could affect the gut microbiota, a community of 100 trillion microbial cells that can enhance human metabolism, immune function, nutrition and other physiological activities [102]. A Chinese research team’s first investigation of HRW, released in January 2018, showed that HRW administration in an animal model affected radiation-induced small intestine toxicity [103]. Ikeda et al. investigated the effects of HRW treatment as preventive measure against bacterial translocation in a murine model of sepsis. Zheng et al. (2018) studied the intestinal microbiota response to 25 d oral administration of HRW and lactulose in female piglets fed *Fusarium* mycotoxin-contaminated maize [104]. The results of this study also showed that HRW treatment affected various intestinal segments, with fewer *Escherichia coli* and more *Bifidobacterium* in the HRW group than in the control group. A 15 d HRW therapy reportedly restored the intestinal barrier that had been damaged by permethrin and increased the amount of butyric acid in the feces. Moreover, a first-in-human trial supported HRW consumption and its positive impact on gut microbiota [105]. According to another study, HRW protected against inflammatory bowel disease (IBD) in an animal model [106]. Following oral administration, HRW demonstrated positive effects by decreasing epithelial cell apoptosis in the small intestine, maintaining the intestinal barrier and tight junctions and restoring the protein expression and distribution of CLDN3 in the small intestine of female piglets fed food contaminated with *Fusarium* toxins [107]. HRW intake improved glucose tolerance that might be decreased in *Bacteroides* levels [108]. Another clinical study reported that drinking alkaline electrolyzed water for two weeks increased *Bifidobacterium* in healthy volunteers [109]. Jin et al. reported that H_2_ released from the gut by hydrogen nanocapsules could induce an abundance of *Akkermansia muciniphila* and reduce metabolic dysfunction-associated fatty liver diseases [110]. However, the gut microbiota may be the major contributors of the biological effects of exogenous hydrogen consumption. Another study revealed that H_2_ saline therapy modulated the abundance of *Bacteroides* and *Lactobacillus* in feces, which might account for the increase in lipid metabolism in mice fed a high fat diet [111]. A previous study reported that acute exercise augments breathing of H_2_ after the lactulose test [112], and the results, corroborated through a recent gut-exercise, implied that colonic bacteria are an endogenous H_2_ source during exercise [113]. The degree of obesity and leanness has a contributory impact on the gut microbiota, and this was observed in the gut flora of bariatric surgery patients [114]. Therefore, HRW might become an upcoming functional water drink that could enhance and adjust endogenous gut microbiota; however, it should be administered as an experimental drink and not suggested for the general population. Interestingly, the function and composition of the intestinal microbiota that routinely produces H_2_ gas fluctuate throughout the day, and the quantity of H_2_ produced varies depending on the person and time of day. One study revealed H_2_S as a new endogenous factor for regulating the circadian clock [115].

## 10. Protective Effects of H_2_ on the Cardiovascular System

The essential gas signaling molecule nitric oxide (NO) can usually be recognized for inducing vasodilatation, reducing the production of superoxide, decreasing inflammation and improving the production of mitochondrial energy. I/R lung damage reduces by ventilation during warm ischemia, ex-vivo infusion and post-transplantation with NO nonheart-beating lung grafts [116]. Carbon monoxide (CO) has a high affinity for the heme prosthetic community in laboratory studies and has also been shown to enhance the graft function in combination with preservation solutions [117,118]. Ohsawa et al. (2008) found that oral H_2_ water prevented the development of atherosclerosis in an apolipoprotein E knockout mouse model [119]. H_2_S is known to induce smooth muscle relaxation, apoptosis, inflammatory response regulation and oxidative stress relief [120]. Although not a gas transmitter, H_2_ is now called a gaseous signal molecule [118]. The advantages are similar to those of NO, CO and H_2_ sulfide (H_2_S), both physiologically and therapeutically [121,122]. Myocardial damage to the mouse caused by radiation was reduced by H_2_ water [123]. Inhalation of H_2_ in a rat model of post-cardiac arrest syndrome also improved survival and functional performance [124]. Researchers have concluded that improved cold-rat ischemia-reperfusion injuries and frequent drinking of H_2_ water could protect beneficiaries from inflammatory heart and aortic allograft degradation [125]. H_2_ is beneficial in terms of toxicity; it shows no cytotoxicity even at high concentrations. For inhalation, high levels of H_2_ gas are defined as high-pressure. In deep-diving gas blends, H_2_ gas is used to prevent the oxidation and thrombosis of arterial gas [126]. Given that H_2_ is an inert and nonfunctional gas in the body, it is understandable that it has no toxic effects. As described above, the inhalation of 1–4% H_2_ gas is highly effective [20]. Basic and clinical research over the past ten years has shown that H_2_ is a major regulatory pathophysiological factor with anti-oxidative, anti-inflammatory and anti-apoptotic effects on cells and organs [127]. Myocardial transmission releases H_2_ through inhalation or injection with [128], injection with H_2_-rich saline [129], drinking H_2_-rich water [127], taking an H_2_-rich bath and increasing the development of intestinal H_2_ through bacteria [130]. Table 2 summarizes the effects of H_2_ on age-related clinical studies in human diseases. 

## 11. Therapeutic Effects of H_2_ on Parkinson’s Disease (PD) and Co-Relation with Intestinal Microbiome

PD of the substantia nigra with extrapyramidal symptoms is a disorder induced by the degeneration and loss of dopamine-producing cells. However, aggregation of α-synuclein in the intestinal mucosa may be caused by oxidative stress produced by macrophages in the luminal wall due to a hyperpermeable intestinal wall, where the intestinal microbiota significantly affects hyperpermeability-induced oxidative stress that may be linked to synuclein pathology in the enteric nervous system in PD [141]. In a study the breath H_2_ concentrations were analyzed in 28 healthy controls and 37 PD patients after consumption of 6 g lactulose [142].

The clinical manifestation of PD is associated with oxidative stress [143]. In addition, studies have been conducted on the involvement of PD with mitochondrial dysfunction [12,143]. Both animal PD models and clinical trials have reported the effects of H_2_ on PD [12]. Oxidative stress was inhibited in the nigrostriatal pathway with the intake of H_2_-rich- drinking water, and the loss of dopamine cells was reduced. These findings indicated that consuming water rich in H_2_ may influence the onset of PD [134]. A randomized double-blind study found that 48 weeks of intake of H_2_-rich water (1000 mL/day) substantially increased the overall Unified PD Rating Scale (UPDRS) score for levodopa-treated PD patients. A double-blind, multicenter H_2_ water study is currently underway [144]. According to one study, intestinal permeability increased in PD, and its level favorably correlated with intestinal staining for *Escherichia coli*, nitrotyrosine and other proteins subjected to protein oxidation [145]. However, another study demonstrated that decreased H_2_ production by the intestinal microbiota is associated with the development and progression of PD [146]. One study also showed how much H_2_ was produced by the seven bacterial strains representing the main bacterial species and groups in the intestine [146]. According to Scheperjans et al., on analysis of the 16S ribosomal RNA genes of the gut microbiota in 72 PD patients and 72 controls, the degree of postural instability was favorably correlated with the relative abundance of Enterobacteriaceae [147].

## 12. Therapeutic and Preventive Effect of H_2_ on AD and Co-Relation with Intestinal Microbiome

The term “gut microbiota” refers to the microbial community that inhabits the gastrointestinal system and may include bacteria, fungi, and protozoans that coexist harmoniously within our intestine [148,149]. This microbiota regulates host homeostasis and many diseases and may play a significant pathogenic role in neurodegenerative disorders, including AD [150,151]. The therapeutic and preventive applications of H_2_ have been confirmed in more animal and human studies, such as in neurodegeneration [152], inflammation [153], and I/R injuries [154]. The gut microbiota, however, has recently been shown to play a significant role in the development of host immunity, in controlling gut endocrine functions and in controlling other neurological signaling [155]. Moreover, the gut microbiome and H_2_ consumption relationship is quite limited. One study found that HRW could improve the structural integrity of the butyrate-producing bacteria in the gut, along with the clinical symptoms of disturbed gut microbiota [156]. Another study demonstrated that HRW intake induced a significant increase in the relative abundance of *Lactobacillus* and a decrease in *Bacteroides* and *Bifidobacterium*. Additionally, because the gut microbiota is important for both health and disease, the impact of HRW on the gut microbiota may greatly improve these diseases. One study revealed that patients with AD showed an increased proinflammatory endobacteria species of *Escherichia coli* and decreased anti-inflammatory taxon, such as *E. rectale*, which may result in microbiota modification, amyloidosis and peripheral inflammation [152].

The deposition of amyloid beta (Aβ) protein outside nerve cells and the accumulated tau phosphorylated protein inside nerve cells are characteristic of the pathology of Aβ protein deposition. Oxidative stress and neuroinflammation have in recent years been documented to be correlated with AD [157]. To date, studies have focused on the role of oxidative stress in the brain parenchyma [158,159]. Aβ protein accumulation is highly linked to the absence of Aβ clearance, which is intricately linked to AD’s pathogenesis [160,161]. It is understood that Aβ protein removal requires low-density lipoprotein receptor 1 (LRP1). The onset of AD involves LRP dysfunction due to oxidative stress and neuroinflammation [161]. The initiation and progression of AD can be prevented by regulating oxidative stress and neuroinflammation. The effect of H_2_ on AD prevention has been investigated in several studies [162]. A rat AD model has been identified in the hippocampus and cerebral cortex; herein, memory was enhanced using H_2_-rich saline (5 mL/kg, i.p., daily) as an inhibitor of oxidative stress, cytokine development and NF-κB production [163]. H_2_-rich water consumption has also been reported to prevent changes in the brain age and decline in spatial memory [164]. Moreover, H_2_ water also exhibited the potential to control dementia at the mild cognitive impairment stage of AD [165]. Safety is a primary concern with respect to H_2_ transportation, storage and administration. H_2_ is flammable only at temperatures greater than 527 °C and explodes by rapid chain reaction with oxygen in the H_2_ concentration range of 4–75% (vol/vol) [166,167]. Because inhaling 1–4% H_2_ has demonstrated great efficacy in medical applications, the use of H_2_ at such low concentrations has been deemed feasible and safe [168].

## 13. Effects of H_2_ in Heart Diseases

Ventricular remodeling contributes to several molecular and cellular pathways in response to pathophysiological stimuli, such as myocardial I/R, hypertension or neurohumoral triggers [169,170]. Endothelin-1 (ET-1) innovations are increased, and Ang II, catecholamines and proinflammatory cytokines activate the receptors and downstream signaling events of their cognates, leading to apoptosis or hypertrophy [169,170]. Until the coronary blood flow was restored in the occluded region, the inhaled H_2_ was rapidly brought into the ischemic myocardial system, and 2% H_2_ was inhaled at the time of ischemia and persisted for 60 min after reperfusion decreased the duration of infarction [169]. In H_2_, for example, the myocardial I/R injury infarction scale is reduced by NO also [169]. In addition to H_2_ inhalation, intraperitoneal saline injection has been shown to reduce the effects of myocardial I/R and also improve heart activity through its anti-oxidant, anti-apoptotic and anti-inflammatory effects [171]. Inhalation of H_2_ at low levels in the C57BL/6J left ventricular myocardial mice (1.3 vol/100 vol) decreased transient dyslipidemia caused by hypoxia, oxidative stress and preventive cardiomyocyte and perivascular fibrosis [145]. Neurohumoral activation, such as β-adrenoceptor and Ang II enhancement, not only results in hypertension, but also leads to ischemic heart diseases as well as sleep apnea syndrome [172]. Direct inhibition of NADPH oxidase expression and decrease in mitochondrial damage leads to ROS inhibition and consequent degradation of the downstream signaling ERK1/2, p38, and JNK, leading to the protective effect of H_2_ [173]. In addition, rats are protected by anti-oxidants and anti-inflammatory activities, such as high-dose ISO-induced acute myocardial infarctions [173]. H_2_-rich saline spontaneously attenuates left ventricular hypertrophy in hypertensive rats by suppressing the inflammatory mechanisms, minimizing oxidative stress, maintaining mitochondrial production, and locally inhibiting Ang II in the left ventricle [174].

## 14. Conclusions

H_2_ is readily available because it has minimal harmful effects and is highly effective against nearly all pathogenic states related to oxidative stress and inflammation. H_2_ has great potential for protective applications in many diseases, owing to its efficacy. Additionally, H_2_ gas has proven to be safe in numerous studies, which is crucial for clinical experiments. H_2_ controls aging primarily through anti-inflammatory and anti-oxidative properties. The treatment of numerous age-related diseases is possible with H_2_ as promising therapeutic and protective options in the future. In addition, H_2_-based therapies are anticipated to be novel and revolutionary methods for the prevention of age-related diseases, thereby promoting helpful longevity.

## Figures and Tables

**Table 1:** Various routes of administration, application and mechanisms of action of H_2_ in cancer treatment.

Route of Administration Category	Application Purpose	Function	Reference
H_2_-rich saline	Cisplatin-induced damage to ovarian cortex	Stimulation of Nrf2 pathway	[86]
	Improvement of cardiac dysfunction caused by Doxorubicin	Inhibition of ROS, Inflammatory cytokines and apoptosis	[88]
H_2_ Pd nanocrystals	synergistic impact with thermal therapy	Provocation of ROS	[89]
H_2_ inhalation	Development of inhibition and improvement of survival rate in glioblastoma	Inhibition of cancer stem cell properties	[90]
	Reversal of renal toxicity due to cisplatin	Inhibition of apoptosis and ROS	[91]
	suppression of tumor growth	Arrest and induction of apoptosis	[92]
H_2_-rich water	Improvement of mFOLFOX6 regimen-induced liver toxicity	Inhibition of oxidative stress	[93]
	44Gy electronic beam reversal of skin damage created	Inflammatory cytokines and oxidative stress reduction	[94]
	inhibition of cancer stem cells	Inhibition of angiogenesis	[92]
	Prevention of gefitinib-induced lung injury	Cytokines inflammatory and oxidative stress reduction	[95]
	Prevention of cisplatin-induced nephrotoxicity	Elimination of oxygen radicals	[73]
	Reversal of mortality and body-weight loss caused by cisplatin	Inhibition of ROS	[73]
	Incidence of tumors and suppression of growth	Inhibition of inflammatory cytokines and oxidative stress, Induction of apoptosis	[96]
	Improved quality of life	Action of antioxidants	[73]

**Table 2:** Effect of H_2_ on age-related clinical studies in human diseases.

Authors	Category of Disease	Sample Size	Route of Administration	Application	Reference
Sakai et al.	Vascular feature of the endothelium	34	Water	Vasomotor activity	[131]
Ostojic et al.	Metabolic acidosis caused by exercise	52	Water	Increased alkalinity of blood in men who are physically active	[132]
Kajiyama et al.	Type II diabetes mellitus	30	Water	Improvement in LDL-cholesterol fractions and glucose tolerance test	[133]
Nakao et al.	Metabolic Syndrome	20	Water	Enhancement of oxidative stress urinary markers	[125]
Yoritaka et al.	PD	17	Water	Improvement of Total Unified PD	[134]
Nakayama et al.	Chronic renal insufficiency	29	Dialysis	Improved markers for inflammation and oxidative stress	[61]
Kang et al.	Adverse effects of radiation-induced liver tumors	49	Water	Improved quality of life ratings during radiotherapy	[87]
Xia et al.	Chronic hepatitis B	60	Water	Reduced oxidative stress	[135]
Ishibashi et al.	Rheumatoid arthritis	20	Water	Improved rheumatoid arthritis activity score	[136]
Aoki et al.	Fatigue in the muscles	10	Water	Improvement in muscle tiredness among young athletes	[137]
Nagatani et al.	Ischemia of the cerebrum	38	Intravenous infusion	Decrease in a subset of patients of MDA-LDL, an oxidative stress serum marker	[138]
Li et al.	Skin pressure ulcer	22	Water	Reduction in wound size and early recovery from skin pressure ulcers	[139]
Ostojic et al.	Soft tissue damage linked to sports	36	H_2_-rich tablets and topical H_2_ packs	Decrease in the viscosity of plasma	[140]

## Abbreviations

8-OHdG	8-hydroxy-desoxyguanosine
AD	Alzheimer’s disease
ATP	Adenosine triphosphate
Aβ	Amyloid beta
BUN	Blood urea nitrogen
DNA	Deoxyribonucleic acid
ET-1	Endothelin-1
GHSR1-α	Growth hormone secretagogue receptor
GSC	Glioma stem-like cell
GSH	Glutathione
H_2_	Molecular Hydrogen
HRW	Hydrogen rich water
H_2_S	Hydrogen sulfide
HMGB-1	High-mobility group box 1
HO-1	Heme oxygenase-1
IL	Interleukin
JNK	c-Jun N-terminal kinase
LRP1	Low-density lipoprotein receptor 1
MDA	Malondialdehyde
MMP3	Matrix metalloproteinase 3
MS	Metabolic syndrome
NO	Nitric oxide
Nrf2	Nuclear factor erythroid 2–related factor 2
PD	Parkinson’s disease
ROS	Reactive oxygen species
SOD	Superoxide dismutase
T2D	Type 2 diabetes
TNF-α	Tumor necrosis factor-α
